# Editorial: The Autophagy Pathway: Bacterial Pathogen Immunity and Evasion

**DOI:** 10.3389/fimmu.2021.768935

**Published:** 2021-09-28

**Authors:** Chinnaswamy Jagannath, Jere W. McBride, Isabelle Vergne

**Affiliations:** ^1^ Houston Methodist Research Institute, Weill Cornell Medical College of Cornell University, Houston, TX, United States; ^2^ Department of Pathology and Institute for Human Infections and Immunity, University of Texas Medical Branch, Galveston, TX, United States; ^3^ Institut de Pharmacologie et de Biologie Structurale, UMR 5089 CNRS - Université de Toulouse, Toulouse, France

**Keywords:** autophagy, immunometabolism, *Mycobacterium tuberculosis*, *Staphylococcus aureus*, *Salmonella typhimurium*, *Listeria monocytogenes*, *Anaplasmataceae*, host-directed therapies

## Overview

Autophagy is a lysosomal degradative pathway which plays important roles in innate immunity against bacterial pathogens (1). Autophagy enables autophagosomes to engulf and deliver intracellular pathogens to the lysosomes for degradation. In addition, autophagy is implicated in the regulation of inflammation by modulating cytokine production. Not surprisingly, bacterial pathogens have developed multiple strategies to manipulate autophagy in order to survive inside the host (2). Although our knowledge of the interplay between bacterial pathogens and autophagy has considerably improved in the past fifteen years, many questions remain. In this Research Topic, we have assembled several research articles and reviews that respond to some of those questions in regard to the host and bacterial factors involved in autophagy regulation, the crosstalk between autophagy and other host defense mechanisms, and the manipulation of autophagy for host-directed therapies.

## Autophagy as a Host Defense Mechanism

The role of autophagy in innate immunity is well conserved across eukaryotic kingdoms. Several vertebrate models such as mice and zebrafish have been essential for our understanding of the role of autophagy, *in vivo*, in bacterial infection (3, 4). Recently, the yellow mealworm beetle (*Tenebrio molitor*) model has also been developed to study *Listeria monocytogenes* infection (Jo et al.). In this model, authors have found a possible dialogue between autophagy and the NF-κB pathway as observed by others in mammalian cells. Autophagy is well known to extensively crosstalk with other innate immune responses including the inflammasome and type I interferon-mediated responses (1). In this Research Topic Paik et al. review the relationship between autophagy and immunometabolism in defense against mycobacterial infection. Both processes appear to be connected by two key kinases, mTOR and AMPK, which regulate TFEB, a central transcriptional factor of autophagy and lysosome machinery. A central role of mTOR in immune responses was further highlighted by Etna et al. who found that rapamycin, an mTOR inhibitor and autophagy activator, modulates expression of regulatory cytokines in *Mycobacterium tuberculosis*-infected dendritic cells. Lastly, Gauron et al. unveiled an important function of another kinase PKCα which inhibits autophagy in the context of *Staphylococcus aureus*. Besides kinases, microRNAs extensively regulate bacterial autophagy (5). Liu et al. found that microRNA-106a dampens autophagy by repressing ULK1, ATG7 and ATG16L1 during mycobacterial infection. Taken together, these articles underscore the multiple roles of host kinases and microRNAs in autophagy regulation and their dialogue with other host defense mechanisms.

## Autophagy Manipulation by Bacterial Pathogens

Several intracellular bacterial pathogens can evade autophagy including *M. tuberculosis*, *L. monocytogenes and Salmonella* typhimurium (2). However, the underlying molecular mechanisms are not fully characterized. Zhou et al. found that *Salmonella* SpvC blocks autophagosome formation through its phosphothreonine lyase activity. Interestingly, Rao et al. observed a reduction of TFEB and lysosomal expression during *Salmonella* infection of macrophages, possibly through caspase-1 activation. Whether SpvC is implicated in that process remains to be investigated. In contrast to Salmonella, other bacterial pathogens exploit autophagy to persist and proliferate in host cells. Patterson et al. review our current knowledge of the interplay between autophagy and *Anaplasmataceae*. Members of this family exploit autophagy to acquire nutrients while avoiding lysosomal degradation. Interestingly, Bechelli et al. report that *Rickettsia australis* triggers Atg5-dependent autophagy to suppress inflammatory cytokines at both transcriptional and post-transcriptional levels, which favors pathogen survival. In non-phagocytic cells, autophagy is essential for intracellular survival of *Staphylococcus aureus* (Gauron et al.). Mulcahy et al. found that *S. aureus* intracellular survival also requires autophagy in primary human neutrophils, although, the specific role of autophagy in such cells was not elucidated. Importantly, the last stage of autophagy is blocked in both phagocytes and non-phagocytes. Overall, these findings bring novel molecular insights on how diverse bacterial pathogens can avoid or, in contrast, use autophagy to persist and flourish in their host.

## Autophagy as a Target for Host-Directed Therapies

With the continuous rise of bacterial multidrug resistance, alternative approaches to combat such pathogens has become a top priority. One promising avenue is to boost host immune responses including autophagy. This approach is particularly relevant for pathogens that are susceptible to autophagy such as *M. tuberculosis* (6). Strong et al. discuss in depth the opportunities and limitations of autophagy-based therapies against mycobacterial infections. The authors have compiled a list of autophagy-inducing compounds that have been tested on cellular and animal models of mycobacterial infection. Notably, some, if not all of these compounds may also modulate other immune responses as Paik et al. and Etna et al. have pointed out. Thus, a comprehensive analysis of the immune responses to infection after treatment with autophagy-inducing molecules may be extremely informative. Importantly, the involvement of autophagy in the control of bacterial infections should be evaluated in detail using *in vivo* models. In conclusion, autophagy appears to be a promising target for treating mycobacterial infections and, thus, it would be worth examining its potential in the context of other bacterial infections.

## Perspective and Future Directions

Overall, this Research Topic highlights the intricate interplay between autophagy and various bacterial pathogens. A better understanding of the role and regulation of autophagy in various cellular niches and relevant animal models, as well as its crosstalk with other host defense mechanisms is essential if one wants to harness autophagy for therapeutic purposes.

## Author Contributions

IV conceived and wrote the first draft of this Editorial. CJ and JM reviewed and edited the manuscript. All authors contributed to the article and approved the submitted version.

## Funding

CJ was supported by NIH RO1 AI-122070 (PI) and seed funds from HMRI, WCM. JM was supported by NIH AI123610, AI126144, and AI149136. IV was supported by Fondation pour la Recherche Médicale (Equipes FRM DEQ20180339208) and the Fondation MSDAvenir (Fight-TB project).

## Conflict of Interest

The authors declare that the research was conducted in the absence of any commercial or financial relationships that could be construed as a potential conflict of interest.

## Publisher’s Note

All claims expressed in this article are solely those of the authors and do not necessarily represent those of their affiliated organizations, or those of the publisher, the editors and the reviewers. Any product that may be evaluated in this article, or claim that may be made by its manufacturer, is not guaranteed or endorsed by the publisher.
